# Supplementary material for: Association between oral microbiome and seven types of cancers in East Asian population: a two-sample Mendelian randomization analysis
Source: Front Mol Biosci. 2023 Nov 21;10:1327893. doi: 10.3389/fmolb.2023.1327893 (PMC10702768; doi:10.3389/fmolb.2023.1327893)
Supplement: Supplementary file 1 [file DataSheet1.ZIP › Supplementary Materials/MR plots of saliva/saliva═╝/Prostate cancer/pheno.2261_to_prostate cancer_forest.pdf]

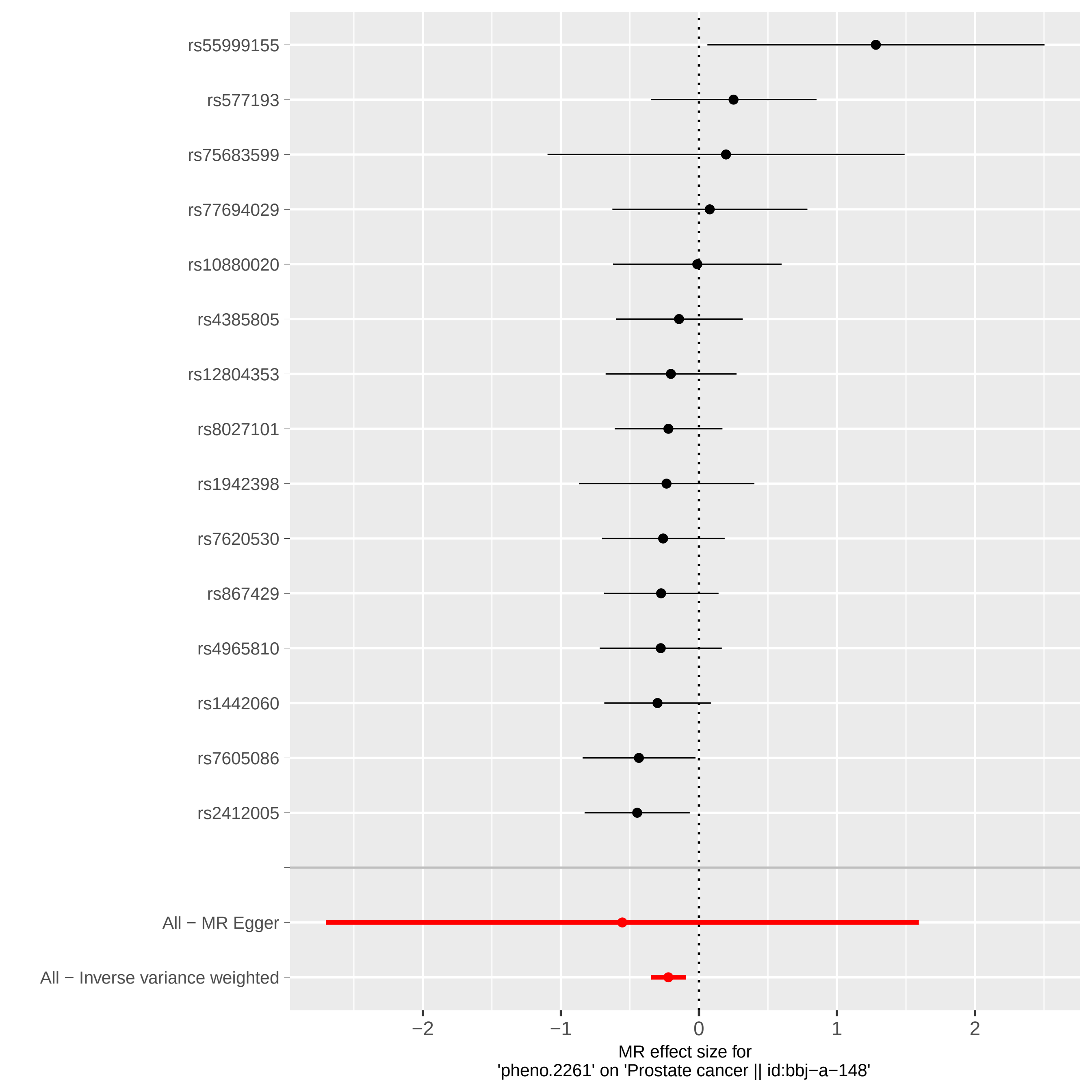
rs55999155
rs577193
rs75683599
rs77694029
rs10880020
rs4385805
rs12804353
rs8027101
rs1942398
rs7620530
rs867429
rs4965810
rs1442060
rs7605086
rs2412005
All − MR Egger
All − Inverse variance weighted
−2
−1
0
1
2
MR effect size for
'pheno.2261' on 'Prostate cancer || id:bbj−a−148'
